# Supplementary material for: Individuals with Type 2 Diabetes Mellitus Tend to Select Low-Carbohydrate, Low-Calorie Food Menus at Home on Diet Application
Source: Nutrients. 2022 Oct 14;14(20):4290. doi: 10.3390/nu14204290 (PMC9610133; doi:10.3390/nu14204290)
Supplement: Supplementary file 1 [file nutrients-14-04290-s001.zip › nutrients-1892613-supplementary.pdf]

nutrients

MDPI

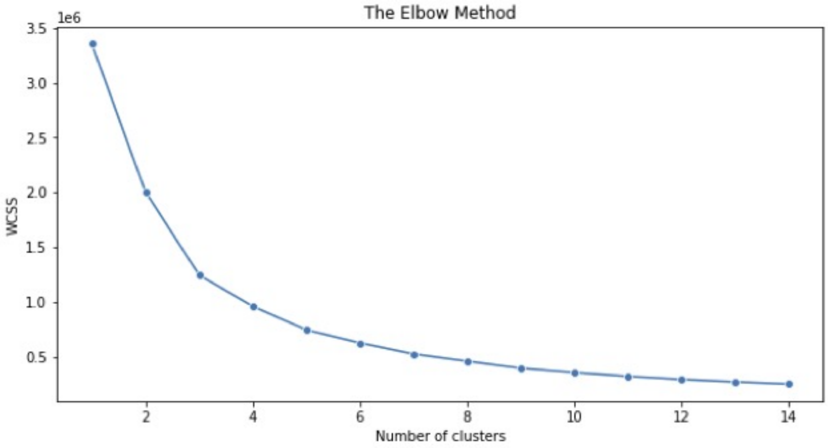

Figure S1: Search for optimal clusters numbers. In the elbow method, the above SSE was calculated by changing the number of clusters, and the results estimate the optimal cluster numbers. K = 11 to 13; clusters 11 to 13 were found to be optimal.

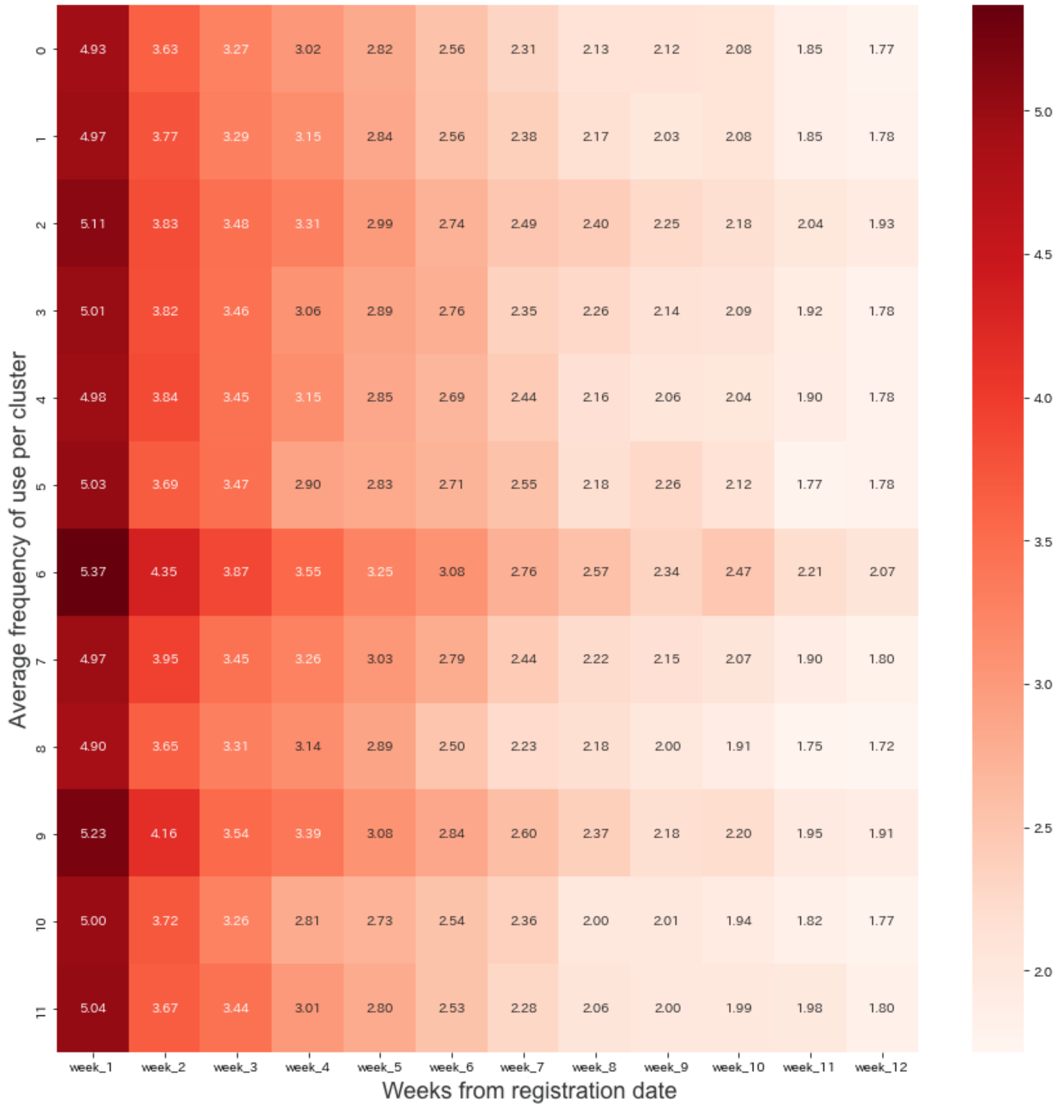

Figure S2: Heatmap of average application usage frequency per cluster. The figure shows the number of times the application was used per week for 12 weeks from the sign-up date. The frequency of use is indicated by a shade of red, with the more frequent the use, the darker the color.

Table S1: Profile of each nutritional cluster

Cluster number	Number of users	Total menu count in cluster	Average of menu count	Average of Age (yo)	Average of height (m)	Average of weight (kg)	Average of BMI (kg/m²)	Average number of days of app use per week in 2018-2019
0	265	679	2.56	41.23	1.64	64.34	23.95	1.67
1	302	907	3.00	42.00	1.65	65.73	23.94	1.63
2	304	748	2.46	40.89	1.63	63.23	23.81	1.82
3	311	596	1.92	40.23	1.64	64.49	23.96	1.82
4	311	905	2.91	40.97	1.64	65.05	23.86	1.65
5	146	421	2.88	40.88	1.63	62.59	23.36	1.86
6	230	540	2.35	40.27	1.64	64.84	24.03	1.96
7	249	472	1.90	41.36	1.64	65.12	24.15	1.72
8	298	720	2.42	41.00	1.65	66.23	24.11	1.71
9	303	761	2.51	40.83	1.64	64.91	24.05	1.71
10	222	638	2.87	40.92	1.63	64.33	23.91	1.69
11	223	605	2.71	40.29	1.62	63.61	24.00	1.64

The table presents the average number of days per week of app use per week in 2018–2019 for users in each cluster.

Table S2: Frequency of application use for each nutritional cluster

Cluster number	Regular user	Occasional user	Very rare user	Total amount
0	36	111	117	264
1	47	134	121	302
2	52	141	111	304
3	56	133	121	310
4	48	142	118	308
5	27	57	62	146
6	44	112	73	229
7	41	118	89	248
8	44	136	115	295
9	52	139	111	302
10	28	95	99	222
11	30	110	81	221

Regular users were defined as users who use the application more than 4 times a week. Occasional users were defined as users who use the application more than once per week but less than 4 times per week. Very rare users were users who use the application less than once per week.

Table S3: Application usage of users belonging to each nutritional cluster

Cluster number	Average number of days of app use (day)	Average number of days of access to menus (day)	Average number of menus used	Average number of menus used per day [unduplicated]	Average number of menus used per day [duplicated]
0	72.3	35.9	84.6	3.23	4.25
1	63.4	34.5	77.1	2.84	3.96
2	70.7	36.0	85.9	3.06	4.16
3	70.2	37.7	82.6	2.89	3.92
4	66.4	33.9	77.5	2.93	3.99
5	72.7	34.7	85.7	3.63	4.68
6	73.7	39.4	95.5	3.05	4.15
7	67.8	34.1	84.9	3.14	4.24
8	65.1	35.7	75.4	2.85	3.90
9	68.5	34.7	82.1	2.96	4.12
10	72.5	34.8	85.5	3.07	3.99
11	64.7	33.4	82.0	3.26	4.26

The Table presents the average number of days accessed, days of access to the menus, menus used, menus used per access day, and menus used per day (without or with duplication), during the study period. The actual number of menu references per day would be closer when the same menu was accessed and counted as duplicates.

Table S4: Comparison of nutritional cluster 5 and nutritional cluster 11

	Average of menu count	Average of Age (yo)	Average of BMI (kg/m^2)	Average number of days of app use per week in 2018-2019
Nutritional cluster 5	2.88	40.88	23.36	1.86
Nutritional cluster 11	2.71	40.29	24.00	1.64
p value	0.79	0.71	0.22	0.73

Nutritional clusters 5 and 11 are not significantly different with regard to each item.
